# Metagenomic analysis of the nasopharyngeal microbiomes and resistomes in asthma, COVID-19 infected, and healthy individuals

**DOI:** 10.3389/fmicb.2026.1729707

**Published:** 2026-01-22

**Authors:** Wisnu Adi Wicaksono, Jonathan Thorsen, Jakob Stokholm, Gabriele Berg

**Affiliations:** 1 Institute of Environmental Biotechnology, Graz University of Technology, Graz, Austria; 2 COPSAC, Copenhagen Prospective Studies on Asthma in Childhood, Copenhagen University Hospital - Herlev and Gentofte, Copenhagen, Denmark; 3 Department of Clinical Medicine, Faculty of Health and Medical Sciences, University of Copenhagen, Copenhagen, Denmark; 4 Department of Food Science, University of Copenhagen, Copenhagen, Denmark; 5 Leibniz-Institute for Agricultural Engineering and Bioeconomy Potsdam (ATB), Potsdam, Germany; 6 Institute for Biochemistry and Biology, University of Potsdam, Potsdam, Germany

**Keywords:** asthma, COVID-19, microbiome, nasopharyngeal microbiome, resistome, shotgun metagenome

## Abstract

**Introduction:**

The nasopharyngeal microbiome presents an important environmental human interface and a window in the fight against chronic diseases like asthma, respiratory infections, and antimicrobial resistance. To identify the microbial structure and function, we designed a pilot study with individuals with asthma, COVID-19 infection, and healthy controls.

**Methods:**

We compare the microbial and resistome profiles of healthy individuals, patients with asthma, and patients with PCR-confirmed COVID-19 using shotgun metagenome sequencing. Additionally, metagenome-assembled genomes were generated to assess the virulence potential of the bacteria identified in the nasopharynx.

**Results:**

We found different patterns in microbial diversity, richness, and structure between individuals with asthma and those who are healthy, but not for those with COVID-19. Our results revealed unexpected insights into the quite diverse nasopharynx resistome encompassing 23 distinct drug classes, mainly based on antibiotic efflux (63.9%) and antibiotic inactivation (24.6%), regardless of the disease state. The majority of the antimicrobial resistance genes (ARGs) confer resistance to multidrug (45%), followed by those genes that confer resistance to aminoglycosides, tetracyclines, polymyxin, beta-lactam, and macrolide-lincosamide-streptogramin. A high proportion of ARGs was associated with various *Pseudomonas* species, which was confirmed by analysing metagenome-assembled genomes. *Pseudomonas brenneri* exhibited the highest number of ARGs and virulence factors, indicating notable pathogenic potential.

**Conclusion:**

The study reveals distinct bacterial community compositions in healthy individuals and individuals with asthma. *Pseudomonadales*, particularly *Pseudomonas* species, contribute to the nasopharyngeal resistome. No association was found between nasopharyngeal resistome profiles and asthma development. Future research may explore airway microbial functions’ influence on asthma development.

## Introduction

The nasopharynx serves as a critical interface between the environment and the human body. The nasopharynx functions as a critical connection between the nasal passages and the respiratory system, serving as the interface for inhaled air, pathogens, allergens, and particulate matter. The nasopharynx is inhabited diverse community of commensal and potentially pathogenic microorganisms, crucial for human health (Flynn and Dooley, 2021). The nasopharyngeal microbiome both plays a key role in modulating immune responses and causes diseases, as respiratory infections often result from initial colonization of the nasopharynx by pathogenic microbes, i.e., *Streptococcus pneumoniae* and *Haemophilus influenzae* (García-Rodríguez and Fresnadillo Martínez, 2002; Yu et al., 2025). Given the exposure of the nasopharynx to environmental agents and immune surveillance, understanding the nasopharynx microbiome provides valuable insights into disease susceptibility, potential therapeutic strategies, and the development of interventions such as probiotics, vaccines, and microbiome-targeted therapies.

Asthma is one of the most prevalent chronic diseases characterized by interactions between genetic and environmental factors. This condition often begins in early childhood, with symptoms such as wheezing, shortness of breath, chest tightness, and coughing, which can vary in intensity over time (Bisgaard et al., 2007). This disease impacts over 350 million children, adolescents, and adults worldwide (García-Marcos et al., 2023), and there is a concerning trend of increasing prevalence on a global scale (GBD 2015 Chronic Respiratory Disease Collaborators, 2017). The immunology of asthma is complex and exhibits significant heterogeneity. Several factors are recognized to be associated with an increased risk of asthma prevalence, including genetics, exposure to tobacco smoke, viral infections, air pollution, obesity, and urbanization (Porsbjerg et al., 2023). Conversely, coronavirus disease 2019 (COVID-19), is caused by severe acute respiratory syndrome coronavirus 2 (SARS-CoV-2), and rapidly disseminated globally (Guan et al., 2020). COVID-19 is an acute respiratory illness that can result in respiratory failure and mortality. Recent research utilizing next-generation sequencing technologies has underscored the importance of the airway microbiome in asthma. For instance, microbial diversity, particularly the presence of *Veillonella* and *Prevotella* in the airways at one month of age, is associated with both local immune mediator profiles and the development of asthma by age six (Thorsen et al., 2019). This aligns with findings from another study indicating that the infant airway microbiome plays a significant role in the transmission of infections to the lower respiratory tract, with early asymptomatic colonization by *Streptococcus* emerging as a strong predictor of asthma development (Teo et al., 2015). Moreover, disruptions or imbalances in both gut and lung microbial composition—referred to as dysbiosis—have been associated with respiratory conditions, including asthma and COVID (reviewed in Ancona et al., 2023; Hufnagl et al., 2020).

Limited information is available regarding the airway microbiome, as assessed through shotgun metagenomics, in the context of asthma and COVID-19. The majority of airway microbiome studies have utilized amplicon sequencing of regions of 16S rRNA for bacterial barcoding (Aho et al., 2015). This technique is often preferred over shotgun metagenome sequencing due to the high prevalence of human DNA in airway samples. However, amplicon sequencing offers limited depth in taxonomic annotation, and does not enable the comparison of functional genes, such as antibiotic resistance genes—“resistome.” The latter indicated a correlation with individuals with asthma, as a previous study identified a higher diversity of antibiotic resistance genes encoded by the gut microbiota in this population (Wilson et al., 2024). Moreover, shotgun metagenomics offers a comprehensive advantage over qPCR by analyzing all variants in a single experiment (Bengtsson-Palme et al., 2017). Shotgun metagenomic sequencing can be employed to detect antimicrobial resistance genes in the nasopharynx, enabling the analysis of correlations between resistome abundance, diversity, and the overall microbial community structure.

Microbial exposure is a significant factor influencing the risk of asthma and COVID-19. Here we used shotgun metagenome sequencing to analyze nasopharyngeal aspirates in individuals from COPSAC_2000_ or COPSAC_2010_, who attended the research unit with an acute airway infection during the COVID-19 pandemic. These were divided into individuals with asthma, those infected with COVID-19, and healthy individuals. We included individuals with COVID-19 to systematically evaluate whether the microbiome of individuals with asthma exhibits a distinct composition compared to those with other respiratory illnesses. To date, the microbiome of nasopharyngeal aspirates in individuals with asthma, COVID-19 infection, and healthy individuals has yet to be analyzed. We specifically address the following questions: (1) Are there any overall differences in microbial diversity and community structure between healthy individuals, individuals with asthma, and those infected with COVID-19? (2) Do the resistome profiles of these groups differ from one another? (3) What are the main contributors to the resistome in the nasopharyngeal microbiomes? This pilot study represents the first shotgun metagenomic analysis of nasopharyngeal microbiomes comparing these groups to determine whether variations in disease state correlate with distinct nasopharyngeal microbiomes.

## Materials and methods

### Subject recruitment and sample collection

For this study, participants were recruited from the COPSAC clinic. All participants and their parents are part of the COPSAC_2000_ (Bisgaard, 2004) or COPSAC_2010_ (Bisgaard et al., 2013) cohorts, and have provided written informed consent. A total of 16 patients were included. These include 8 healthy patients, 4 patients with asthma, and 4 patients with PCR-validated COVID-19 infection. The diagnosis of asthma was physician-diagnosed and performed solely in the research unit according to a previously validated algorithm (Bisgaard et al., 2011). Participants were invited to acute care visits in the research unit during the COVID-19 pandemic, whenever they had airway infections. Here, nasopharyngeal aspirates were obtained using a soft suction catheter passed through the nose into the nasopharynx, following established procedures (Bisgaard et al., 2007). The aspirates were diluted in 1 mL of sterile 0.9% NaCl solution, transported to the laboratory, and stored at −80 °C prior to DNA extraction.

The DNA extraction procedure was conducted using MasterPure™ Complete DNA and RNA Purification Kit (Epicentre), following the manufacturer’s guidelines. Control reagents were extracted and used as a negative control. The extracted DNA was stored at −20 °C until further analysis through shotgun metagenome sequencing. The DNA samples were shipped to Novogene (Cambridge, UK). Novogene conducted DNA library preparation and sequencing using an Illumina NovaSeq 6,000 system, with 2 × 150 bp paired-end sequencing. We did not detect any DNA from the negative control. Moreover, the negative control was determined to have insufficient concentration for library preparation and was subsequently excluded from further processing.

### Processing of sequencing data

The process of quality filtering and trimming of the raw sequence reads was carried out using KneadData v0.12.0.^1^ Trimmomatic v0.39 (Bolger et al., 2014) was used to eliminate Illumina sequencing adaptors and conduct initial quality filtering on raw shotgun metagenomic reads by excluding low-quality reads with a Phred score below 20. Subsequently, the reads were aligned against the reference genome (hg37) using Bowtie2 v.2.4.5 (Langmead and Salzberg, 2012) with default parameters to eliminate human host genome contamination. The filtered reads were then used for further analyses.

### Bacterial community structure analysis

To analyze the structure and diversity of bacterial communities, we use Kraken2 v2.1.2 with the Kraken2 PlusPFP database containing genome references from archaeal, bacterial, viral, protozoa, fungi, plant, and human sources.^2^ This software is used to classify shotgun metagenomic reads (Wood et al., 2019). Following this classification, Bracken v2.6.0 (Lu et al., 2017) was used to estimate species abundances. Subsequently, the resulting bacterial and fungal abundance tables were collapsed to genus-level read counts.

### Antibiotic resistance gene analysis

We used the ARGs-OAP (v3.0) (Yin et al., 2023) to identify and quantify antibiotic resistance genes (ARGs). Clean reads were aligned with the structured ARG database (SARG v3.0) using BLAST+ (v2.12.0) (Camacho et al., 2009), applying the criteria of a similarity threshold of 80%, an e-value of ≤1e−7, and a query coverage of at least 75%. The abundance of ARGs was expressed as the ratio of ARG copies to copies of the 16S rRNA gene, as determined by the ARGs-OAP framework.

### Reconstruction of bacterial metagenome-assembled genomes

We employed a variety of binning techniques, such as Maxbin2 v2.2.7, MetaBAT2 v2.12.1, and CONCOCT v1.1.0, (Alneberg et al., 2014; Kang et al., 2019; Wu et al., 2016) to generate metagenome-assembled genomes (MAGs). DASTool v1.1.1 was utilized to identify MAGs with the highest quality from all binning tools (Sieber et al., 2018). The quality assessment of MAGs, regarding completeness and contamination levels, was conducted utilizing CheckM v1.0.13 (Parks et al., 2015). Only medium-quality MAGs with completeness of more than 50% and contamination levels below 10%, were retained for comparison of metabolic capabilities (Bowers et al., 2017). Dereplication of metagenome-assembled bacterial genomes was done using dRep v2.2.3 (Olm et al., 2017), resulting in a nonredundant set of genomes. Taxonomical information for each MAG was obtained through the Genome Taxonomy Database Toolkit (GTDB-Tk), and phylogenetic trees were constructed using PhyloPhlAn (Asnicar et al., 2020; Chaumeil et al., 2020). The generated MAGs were subsequently analyzed using ABRicate,^3^ to align the contigs with the Comprehensive Antibiotic Resistance Database (CARD) (Alcock et al., 2020) and the Virulence Factor Database (VFDB) (Liu B. et al., 2022) and to detect the presence of ARGs and virulence factors. We defined the presence of antibiotic resistance genes and virulence factors in the genome if the sequences have at least 70% similarity and 70% gene coverage. Abundance profiles of each MAG were calculated using CoverM v0.4.0^4^ with the option -m rpkm, to determine MAG abundance as mapped reads per kilobase per million reads (RPKM).

### Statistical analysis

Statistical analysis and graphical representation were performed in R v4.1.2 using the R packages Phyloseq v1.38.0 and vegan v2.6.4 (McMurdie and Holmes, 2013; Oksanen et al., 2007). Microbial alpha and beta diversity analyses were conducted using normalized data obtained through subsampling to the minimum number of reads. An analysis of variance (ANOVA) was used to identify significant differences (*p* < 0.05) in the diversity of microbial communities and antimicrobial resistance genes (ARGs). Group comparisons were made using a t-test for multiple comparisons with Benjamini–Hochberg false discovery rate correction (FDR adjusted *p* < 0.05). For beta diversity analysis, Bray–Curtis dissimilarity matrices were constructed from normalized datasets. The betadisper function in the vegan package v. 2.6.4 was employed to calculate the distance of each sample from the corresponding group centroid based on the Bray–Curtis distance matrix. These matrix distances were subsequently subjected to permutational multivariate analysis of variance using the Adonis2 function and Bonferroni multiple testing correction (*p* < 0.05) to evaluate the significant effects of disease state on the microbial community and resistome compositions. Lastly, biomarkers at the bacterial genus level using a linear discriminant analysis effect size (LefSe) analysis (Segata et al., 2011) for both healthy individuals and those with asthma. We defined differentially abundant taxa as those with an LDA score greater than 3 and an FDR-adjusted *p* value less than 0.1.

## Results

### Characteristic of the study population

Shotgun metagenomic sequencing was conducted on nasopharyngeal aspirate samples collected from 16 participants (10 males and 6 females), including individuals with asthma, COVID-19 infection, and healthy controls (see Table 1). Among these, 11 participants were from the COPSAC_2000_ cohort and 5 from the COPSAC_2010_ cohort. Samples were collected at acute clinical visits arranged due to symptoms suggestive of COVID-19 infection during the first year of the pandemic. Participants’ ages ranged from 9 to 19 years at the time of sample collection. The average age of individuals with asthma was 10 years, those with COVID-19 infection averaged 12 years, and healthy controls had an average age of 15 years.

**Table 1:** Characteristics of the subjects.

Variables	Subjects with asthma	Subjects with COVID-19	Healthy non-asthmatic
Number of subjects	4	4	8
Gender, female, (%)	50% (*n* = 2)	0% (*n* = 0)	50% (*n* = 4)
Age at sampling, mean (range)	10 (9–11)	12 (10–19)	15 (9–19)
Date of sampling (range)	May–September 2020	December 2020–January 2021	May–October 2020
Cohort, COPSAC_2000_, *n* (%)	0% (*n* = 0)	25% (*n* = 1)	50% (*n* = 4)
Cohort, COPSAC_2010_, *n* (%)	100% (*n* = 4)	75% (*n* = 3)	50% (*n* = 4)

### Nasopharyngeal microbial composition

A total of 1,167,675,803 metagenome reads (min: 46,205,750; max: 95,141,275; average: 72,979,737) were generated and analyzed from all samples (Supplementary Table S1). The analysis of the metagenomes using Kraken2 and Bracken indicated that, on average, 99.1% (range: 97.4–99.6%) of the reads were categorized as human reads, while bacterial and fungal reads accounted for, on average, 0.46% (range: 0.002–1.883%) and 0.015% (range: 0.0003–0.148%), respectively. A total of 7,558 bacterial taxa and 87 fungal taxa were identified from the nasopharyngeal aspirate samples. Our analysis indicates that, although the shotgun metagenome reads are relatively shallow, the rarefaction curve suggests that the current sequencing depth is sufficient to capture the overall diversity present in the samples (Supplementary Figure S1). The removal of low-abundance taxa has been recommended to minimize the risk of false positives in shotgun metagenomic data (Edwin et al., 2024; Garrido-Sanz et al., 2022). We therefore excluded rare bacterial genera (relative abundance <0.01%) and used the resulting data for further analysis, which identified 332 bacterial species and 50 fungal species.

### Bacterial communities exhibited differences between healthy individuals and those with asthma

**Figure 1:**
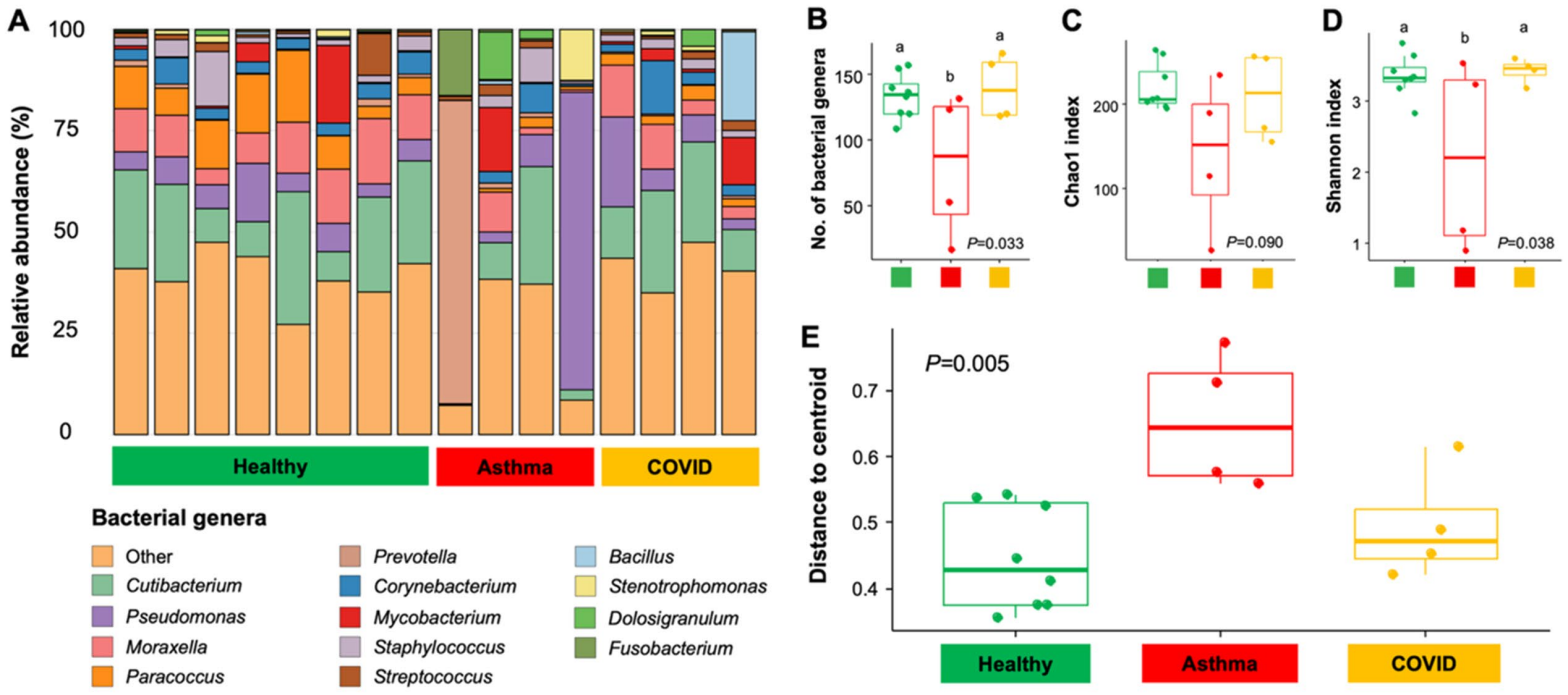
Bacterial community composition and diversity between healthy individuals, individuals with asthma, and individuals infected with COVID-19. A bar plot shows bacterial composition at the genus level **(A)**. Box plots visualize bacterial richness and diversity according to the number of bacterial genera **(B)**, Chao1 **(C)**, and Shannon diversity index **(D)**, respectively. Box plots visualize the variability of bacterial community compositions **(E)**. Each boxplot shows the distance of the respective sample type to the respective group centroid.

The dominant bacteria in the nasopharyngeal aspirates samples belonged to *Actinomycetia* (mean of relative abundance 33.8%), *Gammaproteobacteria* (26.2%), *Alphaproteobacteria* (14.4%), and *Bacilli* (9.1%) whereas the dominant fungi in the samples were *Malasseziomycetes* (42.9%), *Saccharomycetes* (35.3%), and *Sordariomycetes* (11.6%). At genus level, several bacterial genera—including *Cutibacterium*, *Pseudomonas*, *Moraxella*, *Paracoccus*, and *Prevotella*—and fungal genera—including *Aspergillus*, *Fusarium*, *Malassezia*, and *Saccharomyces* —were identified in at least 80% of the samples. These taxa accounted for an average of 46.7% of the total bacterial reads and 87.4% of the total fungal reads. At a more detailed taxonomic level, the bacterial community profiles were primarily dominated by several species, including *Cutibacterium acnes*, *Pseudomonas brenneri*, *Moraxella osloensis*, *Prevotella nigrescens*, *Corynebacterium tuberculostearicum*, *Mycobacterium canetti*, and *Staphylococcus epidermidis*. These species were consistently detected across all samples. Healthy individuals were dominated by bacterial genera *Cutibacterium* (19.2%), *Moraxella* (10.7%), *Paracoccus* (9.6%), *Pseudomonas* (6.4%), *Corynebacterium* (3.7%), and *Staphylococcus* (3.6%). A similar profile was also observed in the individuals with COVID-19. While the bacterial community composition among healthy individuals was relatively similar; a high variation in composition was noted among individuals with asthma. For instance, *Dolosigranulum,* a bacterial taxon typically present in low abundance in healthy individuals, was found in significantly higher abundance in an individual with asthma (Figure 1A). LEfSe analysis identified several taxa as biomarkers that are enriched in healthy individuals relative to those with asthma (Supplementary Table S2). Specifically, *Paracoccus*, *Moraxella* and *Brevundimonas*, were significantly associated with the healthy cohort.

Differences in bacterial community richness and structure were observed between healthy individuals and individuals with asthma. A significantly lower number of bacterial species (S) (*p* = 0.038) and Shannon diversity index (H′) (*p* = 0.016) were observed in individuals with asthma (S = 80, H′ = 2.2) compared to healthy individuals (S = 133, H′ = 3.3; Figures 1B,D). In contrast, no significant difference was detected between the groups when diversity was estimated using the Chao1 index (Figure 1C). In contrast, no significant differences in alpha diversity were observed between the healthy individuals without asthma and individuals with COVID-19. Beta diversity analysis revealed that the disease state explained 20.5% of the bacterial community variation. Multiple pairwise comparisons revealed significantly different clustering of bacterial community structures between healthy individuals and those with asthma (*R*^2^ = 0.204, *P*_adj_ = 0.039), but no significant differences were observed when comparing healthy individuals with those diagnosed with COVID-19 (Supplementary Figure S2). Furthermore, beta dispersion analysis indicated that the variability in bacterial community structure was greater in individuals with asthma compared to their healthy counterparts (Figure 1E).

**Figure 2:**
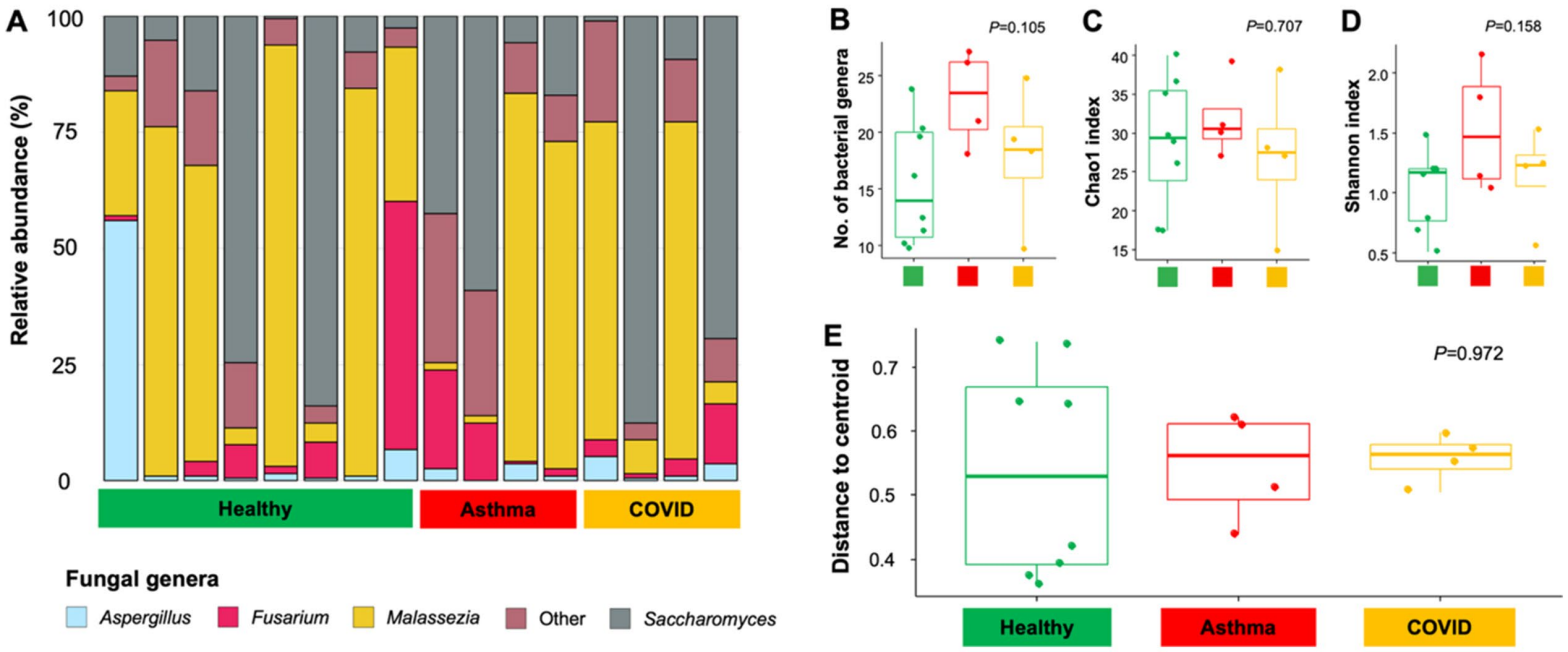
Comparison of fungal community composition and diversity among healthy individuals, individuals with asthma, and those infected with COVID-19. A bar plot illustrating bacterial composition at the genus level **(A)**. Box plots visualize fungal richness and diversity according to the number of fungal genera **(B)**, Chao1 **(C)**, and Shannon diversity index **(D)**, respectively. Box plots visualize the variability of fungal community compositions **(E)**. Each boxplot shows the distance of the respective sample type to the respective group centroid.

Compared to bacterial community profiles, fungal community variation did not exhibit a clear distinction between healthy individuals and those with asthma or COVID-19. For example, the fungal species *Malassezia restricta* was the predominant member of the fungal community in a subset of individuals (*n* = 8), irrespective of their health status (Figure 2A). On the other hand, *Saccharomyces* was the predominant member of the fungal community in other individuals (*n* = 6). A higher number of fungal genera and an increased Shannon diversity index were observed in individuals with asthma compared to healthy individuals and those with COVID-19. However, statistical analysis on fungal data did not reveal any differences in fungal diversity. No differences were observed in the number of fungal genera (*p* = 0.105, Figure 2B), Chao1 index (*p* = 0.707, Figure 2C), and Shannon diversity index (*p* = 0.158, Figure 2D). Similarly, beta diversity and dispersion analysis did not indicate significant differences in fungal community structures (*p* = 0.972) among individuals with various disease states (healthy vs. asthma vs. COVID, Figure 2E). Our findings suggest that the composition of the airway fungal community is more individualized than significantly influenced by the disease state. Consequently, further analyses were concentrated on the bacterial community data.

### The nasopharynx harbors bacterial taxa with multi-antibiotic resistance

**Figure 3:**
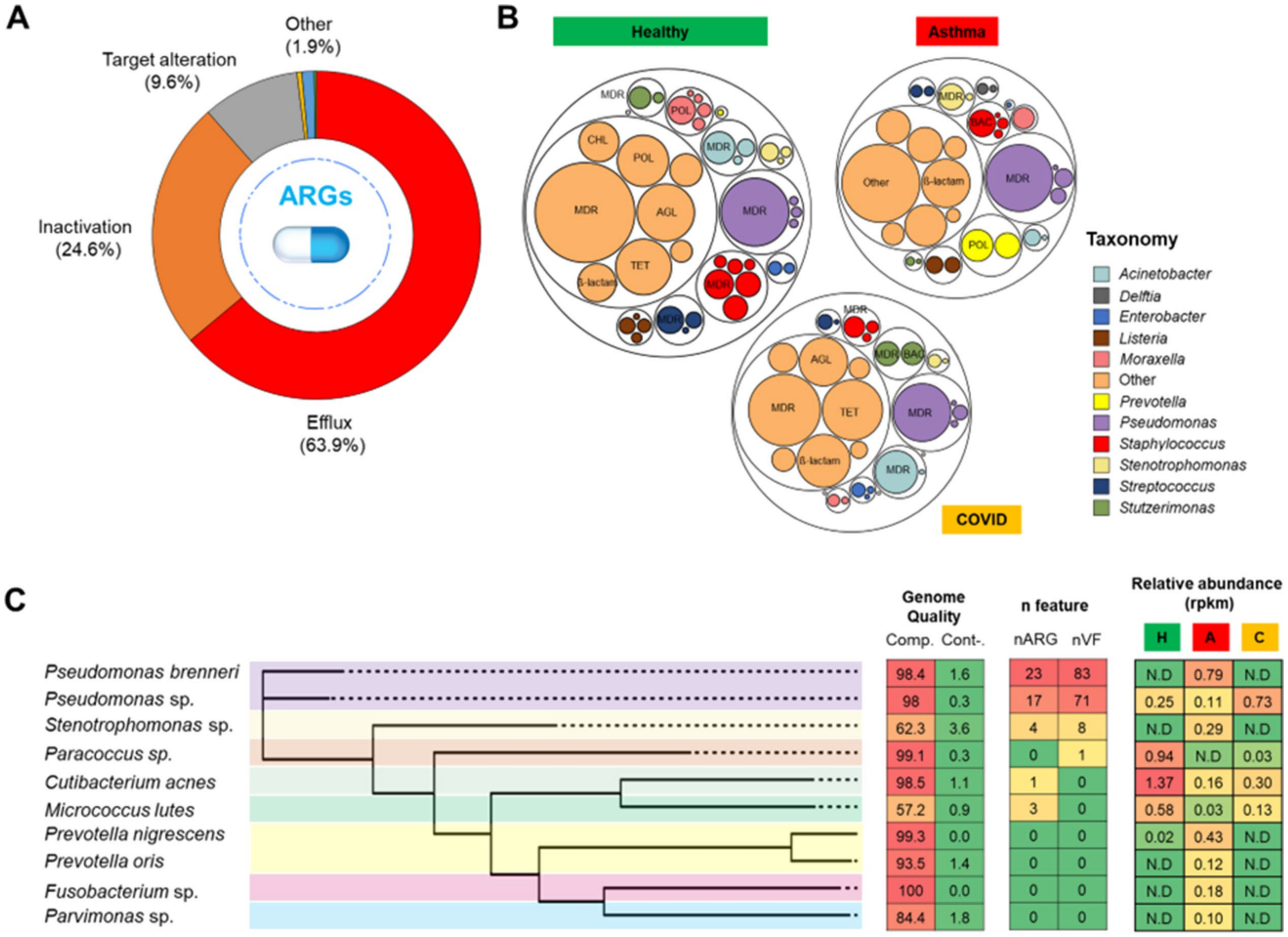
Overview of antibiotic resistance profiles within the nasopharyngeal microbiome. The three primary resistance mechanisms are presented according to their relative abundance within the nasopharyngeal microbiome **(A)**. The resistome profile is derived from the metagenomic dataset. A circle packing plot illustrating antimicrobial resistance gene (ARG) profiling and their bacterial carriers **(B)**. Each bubble represents an ARG class, with bubble size proportional to the number of reads detected in the metagenome. The most abundant ARG classes are labelled. Different colors represent specific antibiotic resistance genes (ARGs) carriers at the genus level. Profiles for selected features, including genome completeness and contamination, number of ARGs, and number of virulence factors (VF), are provided for metagenome-assembled genomes (MAGs) constructed from the nasopharyngeal metagenome **(C)**.

From all samples in the dataset, antibiotic efflux (63.9%) and antibiotic inactivation (24.6%) were the primary mechanisms of resistance observed in the nasopharyngeal microbiome regardless of the disease state (Figure 3A). Collectively, the identified resistance determinants encompassed 23 distinct drug classes. The majority of the ARGs confer resistance to multidrug (45.5%), followed by those genes that confer resistance to aminoglycosides (15.9%), tetracyclines (14.8%), polymyxin (5.3%), beta-lactam (4.9%) and macrolide-lincosamide-streptogramin (3.8%). Furthermore, we assigned taxonomically each read that was annotated as ARGs with Kraken2 (Wood et al., 2019) and identified that a diverse range of taxa contributed to ARG diversity in healthy individuals (Figure 3B). Interestingly, based on the taxonomic classification of the short reads identified as antimicrobial resistance, a high proportion of ARGs (healthy—13.3%, asthma—21.1%, and COVID-19–15%, Figure 3B) were associated with various *Pseudomonas* species, including *P. brenneri*, *P. putida*, *P. aeruginosa*, and *P. fluorescens*. This suggests that this widespread environmental genus was the main contributor to the nasopharyngeal resistome.

We subsequently constructed and analyzed metagenome-assembled genomes (MAGs) to identify potential genes associated with the pathogenicity of taxa linked to disease states. From 16 metagenome samples, we reconstructed 19 bacterial MAGs with a minimum of 30% genome completeness. Additionally, the same bacterial species, such as *Micrococcus luteus* and *Cutibacterium acnes*, were detected across multiple sample (Supplementary Table S3). Of those MAGs, a total of 10 non-redundant MAGs were retained, with at least 50% completeness and less than 10% contamination (Figure 3C). The MAGs were classified within the following taxonomic groups: *Pseudomonas brenneri*, *Pseudomonas* sp., *Prevotella nigrescens*, *Prevotella oris*, *Stenotrophomonas* sp., *Paracoccus* sp., *Cutibacterium acnes*, *Micrococcus luteus*, *Fusobacterium* sp., and *Parvimonas* sp. Notably, 7 of the MAGs were categorized as high-quality, exhibiting at least 90% completeness and less than 5% contamination.

We identified several taxa that harbour multiple antibiotic resistance genes (ARGs) and virulence factors. Most of these taxa belong to the *Gammaproteobacteria* class, namely *Pseudomonas* and *Stenotrophomonas*. The majority of the ARGs detected in these taxa were antibiotic efflux. *Pseudomonas* MAGs that carried multiple ARGs were predominantly detected in individuals with asthma and COVID-19. We conducted further analysis of ARGs and virulence factors within these genomes namely *Pseudomonas brenneri* and *Stenotrophomonas maltophilia* (Supplementary Tables S4, S5). We observed that the MAG identified in this study exhibits profiles similar to those found in host-associated (plant and animal) samples, as opposed to environmental samples. This suggests a potential association with adaptability to the host environment. We also compared the ARG and virulence profile associated with *Stenotrophomonas*, the second taxon containing multiple ARGs and virulence factors, with those of clinical and environmental strains of *Stenotrophomonas maltophilia*, a species known to be an opportunistic pathogen (Supplementary Table S5; Lira et al., 2017). Our analysis revealed a comparatively lower number of detected ARGs and virulence factors in the MAGs identified in this study relative to *S. maltophilia*, suggesting a lower potential pathogenicity of the *Stenotropomonas* characterized herein. We also detected ARGs in MAGs belonging to *Cutibacterium acnes* and *Micrococcus luteus*; however, their numbers were significantly lower than those observed in the aforementioned taxa.

Some MAGs were exclusively detected nasopharyngeal in individuals with asthma. We identified two MAGs belonging to two different species of *Prevotella*: *Prevotella nigrescens* and *Prevotella oris*. Notably, these MAGs were exclusively found in individuals with asthma (Figure 2C). Furthermore, one of the species namely *P. nigrescens* may exhibit increased virulence due to the presence of the *tet37* gene, which confers resistance to tetracycline, along with the virulence factors *gmd* and *neuB.* Virulence factors were also detected from MAGs belonging to *Pseudomonas* and *Stenotrophomonas.* Genes encode different structural parts of the flagellar apparatus, i.e., *flgC*, *flgF*, *flgG*, *flgI*, and *flgG*, and twitching motility phenotype, i.e., *pilG*, *pilH*, and *pilJ* were detected from the MAGs.

### The diversity of antibiotic resistance genes (ARGs) is lower in the nasopharyngeal metagenomes of individuals with asthma

The number of detected ARGs in the nasopharyngeal metagenome samples was different between individuals with different disease states. In our study, nasopharyngeal samples of healthy individuals tend to have a higher number of ARGs in comparison to individuals with asthma and COVID-19 (*p* = 0.093, Supplementary Figure S3A). ARG composition did not show significant variation among individuals with different disease states (*p* = 0.466, Supplementary Figure S3B). The Mantel test indicated a moderate correlation between bacterial composition and the resistome profiles (Mantel test—*p* = 0.016, *r* = 0.487). There were no statistical differences (*p* = 0.387) in ARG load normalized with copy of 16S rRNA genes between different individuals (Supplementary Figure S3C). Consistent results were obtained using RPKM and PPM normalization, with no significant differences in ARG community composition between the tested groups (RPKM—*p* = 0.617; PPM—*p* = 0.677). However, two samples that belong to the asthma group had a relatively lower abundance of ARGs (normalized abundance 0.09 and 0.04) whereas one sample from COVID-19 group had substantially higher ARG load (normalized abundance 5.6) in comparison to other samples (median of normalized abundance 0.56). Subsequent correlation analyses revealed a significant positive association between the number of bacterial taxa belonging to *Pseudomonadales* and the overall ARG load (Pearson’s correlation coefficient—*p* = 0.005, *r* = 0.66), as well as multidrug resistance genes (*p* < 0.001, *r* = 0.78). As mentioned above, our study highlights the significant role of *Pseudomonadales* as the primary contributor to the nasopharyngeal resistome.

We specifically focused on the detection and analysis of genes conferring macrolide resistance. The presence of high-abundance genes conferring resistance to macrolides was previously reported in airway and stool samples from individuals with asthma (Ivan et al., 2024; Wilson et al., 2024) who are often prescribed these medications. However, in our study, this ARG class did not show significant differences among the different disease groups (*p* = 0.636). The aforementioned results indicated that a specific subset of the taxa contributes to the ARG abundance in the nasopharynx. These findings led us to further investigate the correlation between ARG abundance and the diversity and abundance of specific taxa. Subsequent correlation analyses revealed a significant positive association between the number of bacterial taxa belonging to *Pseudomonadales* and the overall ARG load (Pearson’s correlation coefficient—*p* = 0.005, *r* = 0.66), as well as multidrug resistance genes (*p* < 0.001, *r* = 0.78). As mentioned above, our study highlights the significant role of *Pseudomonadales* as the primary contributor to the nasopharyngeal resistome.

## Discussion

The human upper airway harbors a complex microbial ecosystem that plays a critical role in respiratory health, yet its composition and functional potential, particularly in relation to diseseas, remain incompletely understood. Using shotgun metagenomic sequencing of nasopharyngeal aspirates, our study provides a comprehensive characterization of both bacterial and fungal communities as well as antimicrobial resistance genes (ARGs) in healthy individuals and those with asthma and COVID-19. This approach allowed us to identify not only shifts in bacterial community structure associated with asthma but also the key contributors to the airway resistome, including *Pseudomonas* species, and their potential virulence factors.

A low proportion of microbial reads was observed in the nasopharyngeal aspirate samples, consistent with previous reports on the human pharyngeal microbiome. Song et al. (2022) identified that 1.4–4% of total reads from shotgun metagenomic sequencing of nasopharyngeal samples were classified as non-human. In another study, the host DNA content in non-depleted aliquots from nasopharyngeal aspirate samples was found to be 99% (Rajar et al., 2022). Studies on the human pharyngeal microbiome typically use the amplicon sequencing method with specific bacterial and/or fungal primers (Sekaran et al., 2023; Thorsen et al., 2019), which may minimize the sequencing of human DNA. As nasopharyngeal aspirate samples contain a significant amount of human DNA, which can interfere with the detection of microorganisms in metagenomic sequencing, the removal of host DNA may enhance the resolution of microbial DNA (Rajar et al., 2022; Shi et al., 2022). Further validation studies are needed to ensure these strategies are effective without bias.

A notable difference in bacterial community diversity and structure was observed between individuals with asthma and healthy controls, whereas fungal community diversity showed no clear separation between the two groups. A previous study reported that *Moraxella* and *Corynebacterium* were the dominant taxa in the hypopharyngeal microbiota of healthy infants in the first 3 months of life (Mortensen et al., 2016). A high abundance of *Cutibacterium* and *Paracoccus*, has also been reported in nasopharyngeal swabs (Ruiz-Tagle et al., 2023; Sekaran et al., 2023). Moreover, there was a significant presence of *Malassezia* species, specifically *M. globosa* and *M. restricta*, in upper airway samples (Yuan et al., 2023). Compared with healthy individuals, asthma was associated with a shift toward reduced bacterial diversity and dominance of a limited number of taxa, commonly referred to as dysbiosis. Previously, Ivan et al. (2024) identified two asthma-related microbial clusters: one with higher diversity enriched in commensals such as *Prevotella* and *Fusobacterium*, and another with lower diversity dominated by pathogens including *Pseudomonas*. Consistent with this, we observed elevated *Prevotella* and *Fusobacterium* in an individual with asthma, while these taxa were rare in healthy controls (Figure 1). Previous studies have linked *Prevotella*, *Fusobacterium*, and *Dolosigranulum* to asthma (Liu T. et al., 2022; Thorsen et al., 2019; Yang et al., 2022), although their reported associations vary. *Dolosigranulum* was suggested to have a negative rather than positive association with asthma (Zhou et al., 2019) while *Prevotella* has been both positively (Thorsen et al., 2019) and negatively correlated with asthma status (Zhang et al., 2016). Collectively, these findings suggest that decrease in commensal taxa such as *Paracoccus* and *Moraxella* followed by enrichment of taxa such as *Pseudomonas*, *Prevotella*, and *Fusobacterium* in the hypopharynx may contribute to asthma pathogenesis.

In the nasopharyngeal microbiome, ARGs were predominantly associated with multidrug resistance (45.5%), followed by resistance to aminoglycosides (15.9%) and tetracyclines (14.8%), with lower proportions linked to polymyxin, beta-lactam, and macrolide–lincosamide–streptogramin resistance. Data on antibiotic consumption collected from 30 EU/EEA countries over two decades, indicated that members of *β*-lactams, MLS, and tetracyclines classes were among the most frequently consumed antibiotics (Bruyndonckx et al., 2021). Humans frequently encounter specific concentrations of antibiotics during treatment, which can result in the development of the above-mentioned resistance (Pal et al., 2016). Nevertheless, the reasons behind the high prevalence of genes associated with aminoglycoside resistance remain unclear but can probably be explained by the often overlooked environmental dimension of antimicrobial resistance, as bacteria and genes can migrate across different environments (Larsson and Flach, 2022). The majority of bacteria found at this environmental human interface are ubiquitously present in the environment and already equipped with antimicrobial resistance genes, while additional once can be acquired in clinical settings (Mahnert et al., 2019).

Our findings indicate various *Pseudomonas* species, are the predominant contributors to the nasopharyngeal resistome. *Pseudomonas brenneri* exhibited the highest number of ARGs and virulence factors. To our knowledge, *P. brenneri* is not classified as a pathogenic or opportunistic pathogen. This taxon has been identified in a variety of environments, including natural mineral waters, the human oral cavity, plants, and aquatic habitats (Supplementary Table S4). The MAG harbors genes encoding flagellar components and twitching motility proteins which may contribute to its pathogenic potential. In chronic respiratory diseases like cystic fibrosis, *P. aeruginosa* interacts with airway epithelial cells by activating the TLR5 signaling pathway through its virulence factor, flagellin (Ruffin et al., 2021). Recent studies have demonstrated that flagellin may serve as a significant inducer of inflammatory markers, potentially contributing to airway inflammation (Losol et al., 2023). Together, these findings suggest the potential pathogenicity of *Pseudomonas* due to the presence of multiple ARGs and virulence factors. It is important to acknowledge that certain bacterial species may shift from commensal to pathogenic behavior under specific conditions; however, further research is needed to determine if this is true for asthma.

This study presents several limitations. Because the proportion of microbial reads was inherently low despite deep sequencing, any differential findings should be interpreted cautiously, as the dataset may not fully capture low-abundance taxa or ARGs. Our analyses are intended to provide exploratory insights into airway microbial, and resistome profiles. These findings will need to be confirmed in future studies with larger groups of participants. In addition, because this is a small, pilot study from a single site, it has limited ability to detect microbiome differences related to the disease (Walter et al., 2020; Wilson et al., 2024). The shotgun metagenome approach used in this study also does not allow us to assess bacterial viability. Furthermore, some of the differences we did observe may be a result of the age and other characteristics between the participants, which range from children at around 10 years (from COPSAC_2010_) to young adults at about 20 years (from COPSAC_2000_). To better understand the potential role of the airway microbiome in the development of asthma, it may be necessary to enrich the microbial fraction and combine this approach with culturing techniques.

Overall, our study provides evidence of distinct bacterial community compositions among the three tested groups. Healthy individuals exhibit a similar bacterial composition, whereas individuals with asthma show notable differences both from healthy individuals and among themselves. Our findings also suggested that *Pseudomonadales,* particularly various *Pseudomonas* species, are the primary contributors to the nasopharyngeal resistome. However, no association was observed between the nasopharyngeal resistome profiles and the development of asthma. The changes in the bacterial community observed in the hypopharynx across different disease states may provide a basis for future research into how airway microbial functions influence the development of asthma.

## Data Availability

The shotgun metagenome sequencing data have been deposited in the Sequence Read Archive (SRA) under accession number PRJEB92416. All other data supporting the findings of this study, including clinical information, are available from the corresponding author upon reasonable request. Please note that participant-level personally identifiable data are protected under the Danish Data Protection Act and European Regulation 2016/679 (GDPR), which prohibit distribution even in pseudonymized form. However, such data may be made available under a data transfer agreement for collaborative purposes.

## Footnotes

1
https://github.com/biobakery/kneaddata

2
https://benlangmead.github.io/aws-indexes/k2

3
https://github.com/tseemann/abricate

4
https://github.com/wwood/CoverM

## Supplementary material

Supplementary Table S1 Summary of shotgun metagenomic sequencing reads.

Supplementary Table S2 Linear discriminant analysis Effect Size (LEfSE) was conducted to identify differentially abundant bacterial genera between healthy individuals and individuals with asthma.

Supplementary Table S3 Detailed assembly features and taxonomic classification of bacterial metagenome assembled genomes.

Supplementary Table S4 General characteristics of Pseudomonas brenneri isolate and used for ARG and virulence factor profiling.

Supplementary Table S5 General characteristics of Stenotrophomonas maltophilia isolate genomes used for ARG and virulence factor profiling.

Supplementary Figure S1 Rarefaction curves show the number of reads that were classified as bacterial sequences.

Supplementary Figure S2 Hierarchical clustering analysis of bacterial community compositions among individuals with asthma, those infected with COVID-19, and healthy individuals.

Supplementary Figure S3 Antibiotic resistance gene (ARG) diversity and abundance between healthy individuals, individuals with asthma, and individuals infected with COVID-19. A bar plot shows the number of detected ARGs in each sample **(A)**. A principal coordinate analysis (PCoA) plot was used to visualize the clustering of ARG composition **(B)**. A bar plot shows the ARG load, which is presented as copies of ARGs per copy of 16S rRNA genes **(C)**.
