# Supplementary figures and images for: Metagenomic analysis of the nasopharyngeal microbiomes and resistomes in asthma, COVID-19 infected, and healthy individuals
Source: Front Microbiol. 2026 Jan 22;17:1729707. doi: 10.3389/fmicb.2026.1729707 (PMC12872793; doi:10.3389/fmicb.2026.1729707)

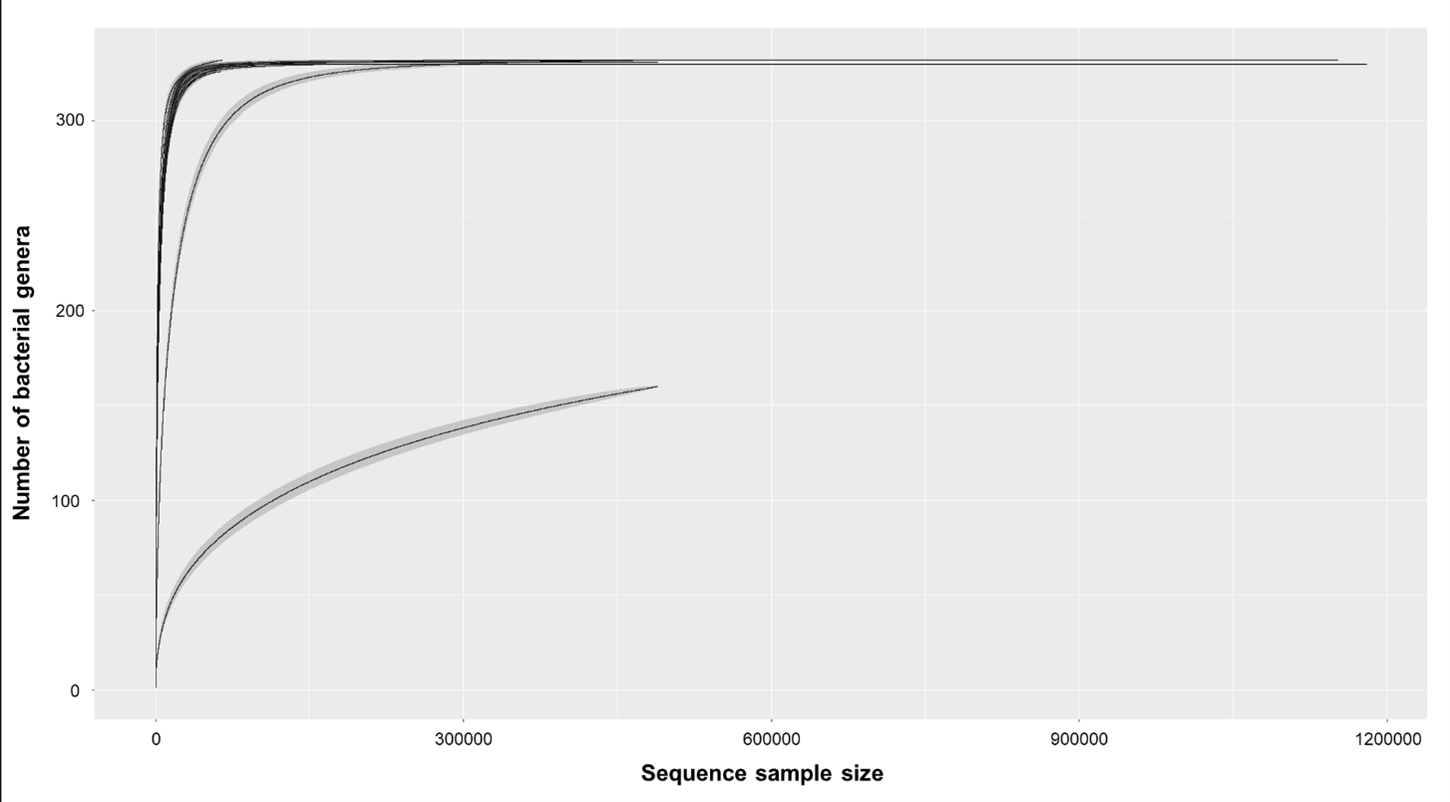

Supplement: Supplementary file 1 [file Image_1.tif]

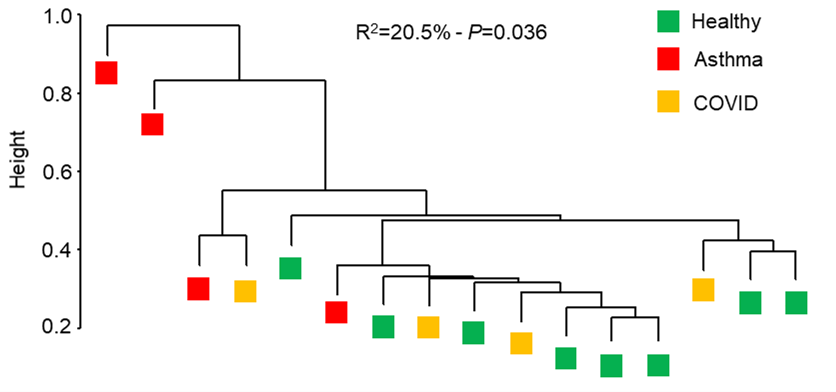

Supplement: Supplementary file 2 [file Image_2.tif]

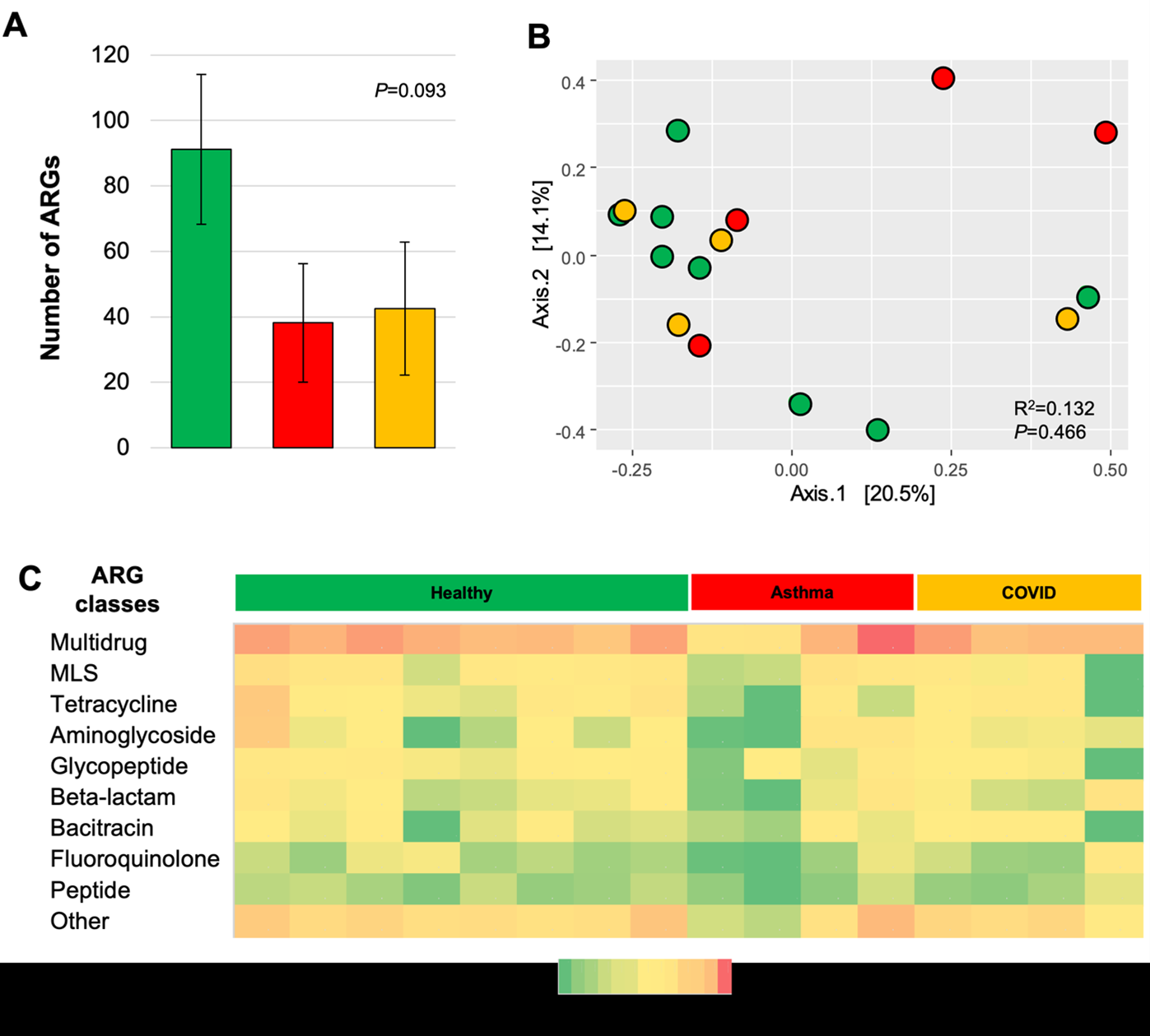

Supplement: Supplementary file 3 [file Image_3.tif]
