# Supplementary material for: Metagenomic analysis of the nasopharyngeal microbiomes and resistomes in asthma, COVID-19 infected, and healthy individuals
Source: Front Microbiol. 2026 Jan 22;17:1729707. doi: 10.3389/fmicb.2026.1729707 (PMC12872793; doi:10.3389/fmicb.2026.1729707)
Supplement: Supplementary file 4 [file Table_1.docx]

**Supplementary Table S1 Summary of shotgun metagenomic sequencing reads.**

| Sample ID | Group | Total high quality reads | Total reads aligned to human genome | Bacterial reads | Fugal reads | ARG reads |
| --- | --- | --- | --- | --- | --- | --- |
| CO1 | Healthy | 65,046,356 | 63,395,983 | 1,208,121 | 96,032 | 67,223 |
| CO4 | Healthy | 83,831,512 | 82,992,583 | 501,861 | 11,053 | 7,204 |
| CO5 | Healthy | 85,145,136 | 84,596,996 | 208,808 | 2,038 | 5,377 |
| CO8 | Healthy | 92,072,571 | 91,757,491 | 3,342 | 590 | 72 |
| CO12 | Healthy | 62,698,885 | 61,149,168 | 1,180,695 | 20,466 | 9,097 |
| CO13 | Healthy | 77,366,513 | 76,997,459 | 97,822 | 5,351 | 1,161 |
| CO14 | Healthy | 62,898,401 | 62,387,370 | 287,046 | 4,992 | 3,948 |
| CO15 | Healthy | 73,289,765 | 72,937,964 | 100,533 | 5,588 | 6,134 |
| CO2 | Asthma | 95,141,275 | 94,319,954 | 491,788 | 253 | 605 |
| CO3 | Asthma | 55,578,123 | 55,373,008 | 1,280 | 193 | 2 |
| CO6 | Asthma | 74,741,745 | 74,414,586 | 65,842 | 1,760 | 1,863 |
| CO7 | Asthma | 75,611,360 | 74,852,863 | 478,730 | 1,527 | 13,271 |
| CO9 | COVID-19 | 66,881,410 | 66,091,345 | 491,696 | 7,124 | 10,623 |
| CO10 | COVID-19 | 77,270,983 | 76,992,296 | 25,404 | 3,802 | 301 |
| CO11 | COVID-19 | 73,896,018 | 73,568,391 | 68,073 | 1,542 | 1,351 |
| CO16 | COVID-19 | 46,205,750 | 46,059,829 | 1,681 | 226 | 50 |
