# Supplementary material for: Metagenomic analysis of the nasopharyngeal microbiomes and resistomes in asthma, COVID-19 infected, and healthy individuals
Source: Front Microbiol. 2026 Jan 22;17:1729707. doi: 10.3389/fmicb.2026.1729707 (PMC12872793; doi:10.3389/fmicb.2026.1729707)
Supplement: Supplementary file 5 [file Table_2.docx]

**Supplementary Table S2 Linear discriminant analysis Effect Size (LEfSE)  was conducted to identify differentially abundant bacterial genera between healthy individuals and individuals with asthma.**

| Bacterial genera | LDA score | Enriched group |
| --- | --- | --- |
| *Paracoccus* | 4.63 | Healthy |
| *Moraxella* | 4.58 | Healthy |
| *Brevundimonas* | 3.99 | Healthy |
| *Acinetobacter* | 3.73 | Healthy |
| *Janibacter* | 3.44 | Healthy |
| *Rhizobium* | 3.07 | Healthy |
| *Roseomonas* | 3.05 | Healthy |
| *Sphingobium* | 3.01 | Healthy |
