# Supplementary material for: Metagenomic analysis of the nasopharyngeal microbiomes and resistomes in asthma, COVID-19 infected, and healthy individuals
Source: Front Microbiol. 2026 Jan 22;17:1729707. doi: 10.3389/fmicb.2026.1729707 (PMC12872793; doi:10.3389/fmicb.2026.1729707)
Supplement: Supplementary file 6 [file Table_3.docx]

**Supplementary Table S3 Detailed assembly features and taxonomic classification of bacterial metagenome assembled genomes**

| Genome ID | Group | comp (%) | cont (%) | dRep cluster | Taxonomy* |
| --- | --- | --- | --- | --- | --- |
| CO1_1 | Healthy | 57.2 | 1 | Cluster 6 | *Actinobacteriota;Actinomycetia;Actinomycetales;Micrococcaceae;Micrococcus;M. luteus* |
| CO1_2 | Healthy | 87 | 0.9 | Cluster 1 | *Actinobacteriota;Actinomycetia;Propionibacteriales;Propionibacteriaceae;Cutibacterium;C. acnes* |
| CO2_1 | Asthma | 84.4 | 1.8 | Cluster 8 | *Firmicutes;Clostridia;Tissierellales;Helcococcaceae;Parvimonas;* |
| CO2_2 | Asthma | 93.5 | 1.4 | Cluster 7 | *Bacteroidota;Bacteroidia;Bacteroidales;Bacteroidaceae;Prevotella;P. oris* |
| CO2_3 | Asthma | 100 | 0 | Cluster 9 | *Fusobacteriota;Fusobacteriia;Fusobacteriales;Fusobacteriaceae;Fusobacterium;* |
| CO2_4 | Asthma | 99.3 | 0 | Cluster 10 | *Bacteroidota;Bacteroidia;Bacteroidales;Bacteroidaceae;Prevotella;P. nigrescens* |
| CO4_1 | Healthy | 86.1 | 1.4 | Cluster 1 | *Actinobacteriota;Actinomycetia;Propionibacteriales;Propionibacteriaceae;Cutibacterium;C. acnes* |
| CO4_2 | Healthy | 44.9 | 10.9 | N.A | *Proteobacteria; Gammaproteobacteria; Pseudomonadales;Moraxellaceae;Moraxella;* |
| CO7_1 | Asthma | 98.4 | 1.6 | Cluster 4 | *Proteobacteria; Gammaproteobacteria; Pseudomonadales;Pseudomonadaceae;Pseudomonas;P. brenneri* |
| CO7_2 | Asthma | 62.3 | 3.6 | Cluster 2 | *Proteobacteria; Gammaproteobacteria; Xanthomonadales;Xanthomonadaceae;Stenotrophomonas;* |
| CO9_1 | COVID19 | 98 | 0.3 | Cluster 3 | *Proteobacteria; Gammaproteobacteria; Pseudomonadales;Pseudomonadaceae;Pseudomonas* |
| CO9_2 | COVID19 | 35.9 | 0 | N.A | *Proteobacteria; Gammaproteobacteria; Pseudomonadales;Moraxellaceae;Moraxella* |
| CO9_3 | COVID19 | 38.41 | 1.1 | N.A | *Actinobacteriota;Actinomycetia;Propionibacteriales;Propionibacteriaceae;Cutibacterium;* |
| CO12_1 | Healthy | 99.1 | 0.3 | Cluster 5 | *Proteobacteria; Alphaproteobacteria; Rhodobacterales;Rhodobacteraceae;Paracoccus;* |
| CO12_2 | Healthy | 98.5 | 1.1 | Cluster 1 | *Actinobacteriota;Actinomycetia;Propionibacteriales;Propionibacteriaceae;Cutibacterium;C. acnes* |
| CO12_3 | Healthy | 40 | 0.4 | N.A | *Actinobacteriota;Actinomycetia;Actinomycetales;Micrococcaceae;Micrococcus;M. luteus* |
| CO12_4 | Healthy | 79.8 | 13.1 | N.A | *Proteobacteria; Gammaproteobacteria; Pseudomonadales;Moraxellaceae;Moraxella;M. osloensis* |
| CO14_1 | Healthy | 78.1 | 1.7 | Cluster 1 | *Actinobacteriota;Actinomycetia;Propionibacteriales;Propionibacteriaceae;Cutibacterium;C. acnes* |
| CO14_2 | Healthy | 36.9 | 2.7 | N.A | *Proteobacteria; Gammaproteobacteria; Pseudomonadales;Moraxellaceae;Moraxella* |

*Taxonomical information – Phylum; Class; Order; Family;Genus; Species

N.A – Not included for MAG dereplication due to genome completeness less than 50% and contamination more than 10%
