# Supplementary material for: Metagenomic analysis of the nasopharyngeal microbiomes and resistomes in asthma, COVID-19 infected, and healthy individuals
Source: Front Microbiol. 2026 Jan 22;17:1729707. doi: 10.3389/fmicb.2026.1729707 (PMC12872793; doi:10.3389/fmicb.2026.1729707)
Supplement: Supplementary file 7 [file Table_4.docx]

**Supplementary Table S4 General characteristics of *Pseudomonas brenneri* isolate and used for ARG and virulence factor profiling**

| *P. brenneri* isolates/ ID | GenBank assembly | Origin | nARG | nVF |
| --- | --- | --- | --- | --- |
| JCM 13307 | GCA_014646715.1 | Environment - Natural mineral water | 22 | 103 |
| CCUG 51514 | GCA_008386555.1 | Environment - Natural mineral water | 22 | 103 |
| DSM 15294 | GCA_007858285.1 | Environment - Natural mineral water | 22 | 103 |
| ERR9968715_bin.6 | GCA_963521945.1 | Human - oral cavity | 19 | 81 |
| M3 | GCA_049199265.1 | Plant - Blueberry stem | 20 | 82 |
| JD2-26 | GCA_041698325.1 | Environment - incinerating facility | 20 | 84 |
| K5-sn1400 | GCA_030370465.1 | Environment - Wastewater | 22 | 85 |
| Ps-23 | GCA_015643585.1 | Animal - Fish *Oncorhynchus mykiss* | 22 | 85 |
