# Supplementary material for: Metagenomic analysis of the nasopharyngeal microbiomes and resistomes in asthma, COVID-19 infected, and healthy individuals
Source: Front Microbiol. 2026 Jan 22;17:1729707. doi: 10.3389/fmicb.2026.1729707 (PMC12872793; doi:10.3389/fmicb.2026.1729707)
Supplement: Supplementary file 8 [file Table_5.docx]

**Supplementary Table S5 General characteristics of *Stenotrophomonas* maltophilia isolate genomes used for ARG and virulence factor profiling**

| *S. maltophilia* isolates | GenBank assembly/Accession | Origin | nARG | nVF |
| --- | --- | --- | --- | --- |
| K279a | GCA_000072485.1 | Clinical - blood | 15 | 13 |
| EA63 | NEQP00000000 | Environment - sewage | 14 | 14 |
| EA21 | NEQQ00000000 | Environment - sewage | 15 | 13 |
| EP5 | NEQR00000000 | Environment - rhizosphere | 10 | 13 |
| EP20 | NEQS00000000 | Environment - rhizosphere | 8 | 12 |
| EA23 | NEQT00000000 | Environment - eye care solution | 15 | 13 |
| PS5 | NEQU00000000 | Environment - rhizosphere | 10 | 13 |
| EA1 | NEQV00000000 | Environment - brackish water | 15 | 16 |
| EA22 | NEQW00000000 | Environment - sewage | 14 | 14 |
| EP13 | NEQX00000000 | Environment - rhizosphere | 15 | 13 |
| NS26 | NEQO00000000 | Environment - dune soil | 15 | 13 |
| E539 | NEQZ00000000 | Clinical - wound | 14 | 13 |
| E539 | NERA00000000 | Clinical - urine | 10 | 14 |
| E861 | NERB00000000 | Clinical - sputum | 15 | 13 |
| D388 | NERC00000000 | Clinical - urine | 15 | 13 |
| E301 | NERD00000000 | Clinical - urine | 9 | 14 |
| G51 | NERE00000000 | Clinical - blood | 14 | 14 |
| E999 | NERF00000000 | Clinical - respiratory secretion | 17 | 14 |
| E759 | NERG01.1 | Clinical - urine | 14 | 15 |
